# Mitochondrial Dysfunction Increases Oxidative Stress and Decreases Chronological Life Span in Fission Yeast

**DOI:** 10.1371/journal.pone.0002842

**Published:** 2008-07-30

**Authors:** Alice Zuin, Natalia Gabrielli, Isabel A. Calvo, Sarela García-Santamarina, Kwang-Lae Hoe, Dong Uk Kim, Han-Oh Park, Jacqueline Hayles, José Ayté, Elena Hidalgo

**Affiliations:** 1 Oxidative Stress and Cell Cycle Group, Universitat Pompeu Fabra, Barcelona, Spain; 2 Pombe Deletion Project, KRIBB, Yuseong-gu, Daejeon, Republic of Korea; 3 Bioneer Corporation, Daedeok-gu, Daejeon, Republic of Korea; 4 Cancer Research U. K., London, United Kingdom; Universität Heidelberg, Germany

## Abstract

**Background:**

Oxidative stress is a probable cause of aging and associated diseases. Reactive oxygen species (ROS) originate mainly from endogenous sources, namely the mitochondria.

**Methodology/Principal Findings:**

We analyzed the effect of aerobic metabolism on oxidative damage in *Schizosaccharomyces pombe* by global mapping of those genes that are required for growth on both respiratory-proficient media and hydrogen-peroxide-containing fermentable media. Out of a collection of approximately 2700 haploid yeast deletion mutants, 51 were sensitive to both conditions and 19 of these were related to mitochondrial function. Twelve deletion mutants lacked components of the electron transport chain. The growth defects of these mutants can be alleviated by the addition of antioxidants, which points to intrinsic oxidative stress as the origin of the phenotypes observed. These respiration-deficient mutants display elevated steady-state levels of ROS, probably due to enhanced electron leakage from their defective transport chains, which compromises the viability of chronologically-aged cells.

**Conclusion/Significance:**

Individual mitochondrial dysfunctions have often been described as the cause of diseases or aging, and our global characterization emphasizes the primacy of oxidative stress in the etiology of such processes.

## Introduction

Reactive oxygen species (ROS) homeostasis plays an important role in chronological aging processes and some degenerative diseases [1]–[5]. The main source of ROS in most cell types is the mitochondria, where they are formed upon incomplete reduction of oxygen at several sites on the electron transfer chain [6]. Physiological ROS levels are achieved by means of a complex collection of cellular activities such as superoxide dismutases, peroxiredoxins, glutathione peroxidases, or catalases. While ROS, such as superoxide or hydrogen peroxide (H_2_O_2_), are mediators of oxygen toxicity, they are also involved in intracellular signaling through the activation of response pathways (for reviews, see 7,8).

Thus, in eukaryotic microbes, ROS sensors and other pathway components have been identified and characterized by treatment of cultures with extracellular peroxides, which rapidly increase the intracellular steady-state levels [8]. Thus, in *Schizosaccharomyces pombe*, the Pap1 and Sty1 pathways quickly respond to moderate and high extracellular concentrations of H_2_O_2_, respectively [9], [10]. These cellular H_2_O_2_ receptors are then able to trigger signaling pathways with the aim of engaging suitable cellular responses, which normally include peroxide scavengers and repair activities, and promote adaptation and survival [10]–[13].

However, little is known about the effect of endogenously induced fluctuations of H_2_O_2_ in microbial model systems or about the effect of changes on the respiratory quotient (rate of carbon dioxide production to oxygen consumption) with regard to intrinsic oxidative stress and damage. Budding yeast suffers glucose-mediated repression of respiration, also known as the Crabtree effect [14]; therefore, this species is known as “fermenting yeast”. Only at the end of the exponential growth phase, when glucose is exhausted, does *S. cerevisiae* start to respire the ethanol generated during fermentation. Whereas sugar utilization by this yeast has been widely studied, little is known about the so-called nonconventional yeasts (for a review, see 15). Unlike budding yeast, *S. pombe* was initially described as a “petite-negative” yeast (requiring mitochondria for growth even in standard glucose-containing media) [16]. It was soon reported that, contrary to other “petit-negative” yeasts [17], [18], *S. pombe* was able to grow under anaerobic conditions and showed repression of respiratory enzymes by glucose [19]. Hence, *S. pombe* is not able to oxidize the ethanol produced during the exponential phase as a sole carbon source, since it lacks a functional glyoxylate cycle [20] . However, respiration of the glucose leftovers is also triggered at the beginning of the stationary phase, with a decrease in the respiratory quotient [19] and an increase in respiratory enzyme levels [21] in the glucose-derepressed early stationary phase. Therefore, according to the literature, *S. pombe* seems to behave very similarly to *S. cerevisiae* regarding glucose utilization, with low respiratory activities during the exponential phase of growth in the presence of fermentable glucose as the carbon source.

We decided to first characterize the metabolism of fission yeast when grown in the two most common glucose-containing laboratory media, defined medium and complex medium. We show that fission yeast grown in glucose-containing defined medium (2% glucose) not only ferments the sugar, but also respires it, as revealed by oxygen consumption measurements. In contrast, respiration is comparatively weaker in cells grown in complex medium, which contains a higher concentration of glucose (3%). To identify genes required for survival upon both exogenous and endogenous oxidative stress, we tested an *S. pombe* collection of viable open reading frame deletion mutants with a double screening procedure. Yeast mutants that are sensitive to growth under respiratory-prone conditions (defined medium) and to H_2_O_2_ on low-respiratory medium (complex medium) may carry mutations in genes that participate in the generation of respiration-linked basal oxidative stress. Out of a collection of approximately 2700 haploid yeast deletion mutants, 51 were sensitive to both conditions and 19 of these were related to mitochondrial function. The basis of double sensitivity to intrinsic and extrinsic oxidative stress of many genes coding for electron transfer chain components is unraveled here.

## Results

### Exponentially growing fission yeast cells respire in fermentable carbon sources at different rates depending on the growth medium

**Figure 1:**
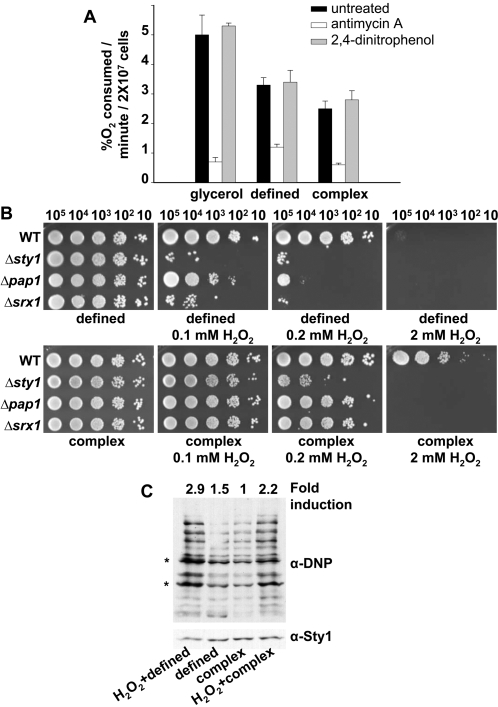
Respiratory rates, intracellular ROS levels and ROS-mediated intrinsic oxidative stress depend on the type of growth media. (A) Oxygen consumption of *S. pombe* cells grown in different growth media. Wild-type strain 972 was grown on glycerol (defined medium with 2% glycerol and 0.2% glucose) (glycerol), defined medium (contains 2% glucose) (defined), and complex medium (contains 3% glucose) (complex), in the presence or absence of the mitochondrial inhibitors antimycin A or 2,4-dinitrophenol. Error bars of this figure represent the standard error measurement (SEM) of three replicates. (B) Survival of different strains in response to H_2_O_2_ exposure in defined *vs.* complex media plates. Strains 972 (WT), AV18 *(Δsty1*), AV25 *(Δpap1*), and EA38 (*Δsrx1*) were grown in defined media, and the indicated number of cells were spotted onto defined or complex media plates with or without H_2_O_2_ at the indicated concentrations. (C) Protein carbonylation generated during growth in defined and complex media. Wild-type strain 972 was grown aerobically in defined or complex media, and cells were collected before or after treatment for 30 min with 2 mM H_2_O_2_ at an OD_600_ of 0.5. Protein carbonylation was detected by reaction of carbonyl groups with DNPH, followed by SDS-PAGE and Western blot analysis by using anti-DNP (α-DNP, top panel) or anti-Sty1 antibodies as a loading control (α-Sty1, bottom panel). Fold induction numbers, obtained from the same blots, are the ratio of the absolute scan numbers for the indicated bands (*) and the corresponding amount of Sty1, and they relate to the values of the growth in complex medium. Similar results were obtained from 7 independent experiments.

In order to verify whether glucose repression of respiration (the Crabtree effect) is severe in *S. pombe* grown in the two most common glucose-containing laboratory media, defined medium and complex medium [22], we measured the oxygen consumption (%O_2_, an indicator of respiratory rates) of cells growing exponentially in defined or complex media using an oxygen electrode, and compared the values to the oxygen consumption of cells growing in non-fermentable glycerol as a carbon source, where oxygen consumption has to be at a maximum. As observed in Figure 1A, exponentially growing *S. pombe* cells respire at different rates depending on the growth media. In all three cases, oxygen uptake was due to normal respiration, since it was abrogated by the complex III inhibitor antimycin A [23], but it was not affected by the uncoupler 2,4-dinitrophenol, which is not expected to decrease oxygen consumption [19] (Figure 1A). Thus, in *S. pombe* oxidative phosphorylation is the main source of energy in glycerol-containing defined medium, but it also occurs in cells growing in glucose-containing defined medium and, to a lesser extent, in complex medium.

### Respiratory rates positively determine the level of intrinsic oxidative stress in fission yeast

The differences in respiratory rates suggested that cells grown in defined medium could have higher levels of intracellular ROS and, consequently, display exacerbated sensitivity to extracellular peroxides when compared with cells grown in complex medium. We measured cell survival against extracellular H_2_O_2_ of wild-type and H_2_O_2_-sensitive cells (eg, cells lacking Pap1, Sty1 or Srx1, a protein essential for basal H_2_O_2_ scavenging) [24] on defined *versus* complex media plates. As expected, cells are more sensitive to extracellular peroxides when grown in defined medium (compare the same concentrations of H_2_O_2_ in complex *vs.* defined media, Figure 1B). In fact, activation of the Pap1 pathway, which detects very low extracellular H_2_O_2_ stress, requires lower concentrations of peroxide in cells grown in defined medium than in complex medium, as shown by the formation of the active, oxidized form of Pap1 (Figure S1A), and by Northern blot analysis of Pap1-dependent genes (Figure S1B). Furthermore, oxidative damage is stronger in wild-type cells grown in defined medium than in complex medium: protein carbonylation in extracts from cells grown in defined medium (Figure 1C, second lane), is 1.5-fold higher than protein carbonylation in extracts from cells grown in complex medium (Figure 1C, third lane).

### Identification of the electron transfer chain as a major determinant of intrinsic oxidative stress

**Figure 2:**
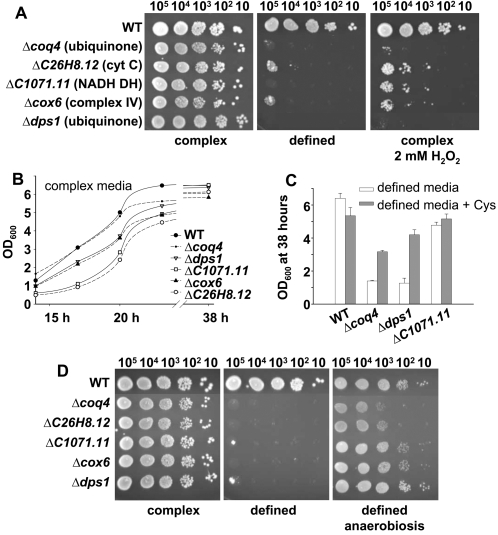
Growth defects of mitochondrial mutants in defined medium are alleviated by the use of cysteine or the absence of oxygen. (A) The growth of some mitochondrial mutants is inhibited by both extracellular H_2_O_2_ and defined medium. Wild-type strain 972 (WT), and the mitochondrial mutants *Δcoq4* and *Δdps1* (ubiquinone), *ΔC26H8.12* (cyt c), *Δcox6* (complex IV), and *ΔC1071.11* (NADH DH) (see Text S1 for details), were grown in liquid complex media, and 10–10^5^ cells were spotted onto defined or complex media plates with or without 2 mM H_2_O_2_. (B) Growth curves of wild-type and mitochondrial mutants in liquid complex medium. Strains such as those used in Figure 2A were grown in complex medium and the OD_600_ were recorded at the times indicated. (C) Maximum OD_600_ reached by wild-type and mutant strains at stationary phase in defined medium in the presence or absence of cysteine. Wild-type strain 972 (WT) and the strains *Δcoq4*, *Δdps1* and *ΔC1071.11* were grown in defined media supplemented or not with 0.1 mg/ml cysteine (Cys) and the OD_600_ was recorded at 38 hours for each culture. Error bars of this figure represent the SEM of five replicates. (D) Survival of wild-type and mitochondrial mutant strains under aerobic *versus* anaerobic conditions. Strains such as those used in Figure 2A were grown in complex medium, and 10–10^5^ cells were spotted onto plates of complex medium, defined medium, or defined medium under anaerobic conditions.

We then designed a double screen to isolate genes required for survival at elevated levels of intracellular ROS. As a first screening step, we checked an *S. pombe* collection of approximately 2700 haploid yeast deletion mutants for strains that were sensitive to growth on defined media plates, where respiratory rates are higher, but not on complex media plates. However, many of the selected mutants would be unable to grow on defined medium due to fundamental problems in biosynthetic pathways. Therefore, and to identify those genes whose inactivation confers sensitivity to intrinsic oxidative stress (eg, by enhancing ROS production or impairing ROS scavenging), our second screening step was the inability to grow on H_2_O_2_-containing complex media plates. We hypothesized that some mutant strains that were sensitive to extracellular peroxides in complex media would also be unable to cope with the oxidative metabolism of growth in defined medium and, therefore, that some genes would be equally necessary for survival in the face of intrinsic and extrinsic oxidative stress. From a total of 51 deletion strains isolated following the double screening procedure, 19 of the deleted genes were involved in different mitochondrial functions (Table 1), from which 12 coded directly for components of the electron transfer chain. We confirmed that none of these mutants could grow on glycerol where respiration is the only energy source (data not shown). Several genes that were unrelated to the mitochondrial category were also isolated in the screen and will be described elsewhere (Table S1). We selected some of the mitochondrial mutant strains for further analysis: *Δcoq4* and *Δdps1* (coding for proteins required for the biosynthesis of coenzyme Q), *ΔC26H8.12* (coding for a protein that catalyzes the cytochrome c-heme linkage), *Δcox6* (coding for the subunit VI of cytochrome c oxidase, or complex IV), and *ΔC1071.11* (coding for a protein containing a flavin reductase-like domain; putative NADH oxidoreductase). To confirm their double sensitivity to intrinsic and extrinsic oxidative stress, we compared the growth of these mutants and a wild-type strain in complex, defined, and H_2_O_2_-containing complex media plates (Figure 2A). To differing degrees, they all showed sensitivity to growth in defined media and in H_2_O_2_-containing complex media.

**Table 1:** Mitochondrial genes required for survival upon extrinsic (extracellular H_2_O_2_) and intrinsic (growth on defined medium) oxidative stress.

NAME	FUNCTION
*C26H8.12*	Covalently links the heme group to the apoprotein of cytochrome c
*C1071.11*	NADH dependent oxidoreductase; contains a flavin like domain
*rip1*	Subunit of cytochrome bc1, also known as respiratory complex III
*sco1*	Copper chaperone protein, essential for complex IV assembly
*C1672.04c*	High similarity to *S. cerevisiae* Cox19p, which is a metal transporter for complex IV assembly
*dps1*	Decaprenyl diphosphate synthase, required for ubiquinone biosynthesis
*cox6*	Heme A-containing chain of cytochrome c oxidase
*coq2*	Required for ubiquinone biosynthesis
*coq3*	Hexaprenyldihydroxybenzoate methyltransferase. Ubiquinone biosynthesis
*coq4*	Ubiquinone biosynthesis protein
*coq5*	C-methyltransferase, ubiquinone biosynthetic process and aerobic respiration
*coq10*	Electron transport and cellular respiration; ubiquinone biosynthesis
*C336.13c*	Removal of transit peptides for the targeting of proteins from the mitochondrial matrix
*tom70*	Receptor that accelerates the import of all mitochondrial precursor proteins
*C8C9.06c*	Mitochondrial translation regulator, PPR domains
*mss1*	GTPase involved in the 5-carboxymethylaminomethyl modification of mitochondrial tRNAs
*C2G2.07c*	Mitochondrial ribosomal protein (small subunit)
*C25B2.04c*	Mitochondrial ribosome assembly protein
*C1610.02c*	Mitochondrial ribosomal protein L1

### The growth defects of respiratory mutants in glucose-containing defined media are alleviated by antioxidants

The growth of these mutants in liquid defined media, but not in liquid complex media, was also severely impaired compared to a wild-type strain (Figure 2B and 2C). Addition of the glutathione-precursor agent cysteine (Figure 2C) or the antioxidant N-acetylcysteine (data not shown) to the liquid defined media enabled their growth to recover, indicating that ROS production may be enhanced in these mutants. Furthermore, incubation of defined media plates under anaerobic conditions partially or totally restored the growth of these mutants (Figure 2D). These data demonstrate that growth impairment of these mutants in defined media is due to intrinsic oxidative stress.

### Mutations in electron transfer chain components increase the levels of intracellular ROS

**Figure 3:**
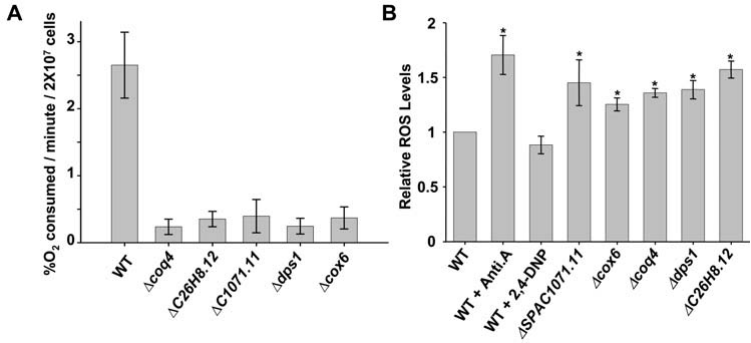
Mitochondrial mutants display reduced oxygen consumption and increased intracellular ROS levels. (A) Oxygen consumption of wild-type and mitochondrial mutants grown in complex media. Strains such as those in Figure 2A were grown in complex medium and oxygen consumption was measured. (B) Relative intracellular H_2_O_2_ levels of wild-type and mitochondrial mutants. Wild-type and mutant strains were grown in complex medium. When indicated, antimycin A (Anti.A) or 2,4-dinitrophenol (2,4-DNP) was added or not to the wild-type cultures. The cells were incubated with DCFH-DA for 40 min and analyzed by flow cytometry, as indicated in Materials and Methods. Significant differences between wild-type and drug-treated or mutant cells were determined by the Student's *t*-test. *p<0.05 compared with untreated wild-type cells. Data in both panels were obtained from three independent experiments and are expressed as mean±SEM.

The requirement of antioxidants or anaerobiosis for growth of the mitochondrial mutants on defined medium suggested that they are more prone to produce ROS than a wild-type strain. Oxygen consumption, and therefore respiration, was severely inhibited in the mutants, as expected (Figure 3A). We tested the steady-state levels of ROS in these mutants by measuring oxidation of the dye 2′,7′-dichlorodihydrofluorescein diacetate (DCFH-DA) (Figure 3B). The levels of fluorescence, which are an indicator of the intracellular concentration of peroxides, during growth in complex media were between 1.3- and 1.7-fold higher in the respiration-deficient mutants than in the wild-type strain (Figure 3B). We propose that electron leakage is exacerbated in those mutants, as described when cells have been treated with electron transfer chain inhibitors such as antimycin A, where accidental ROS production can be enhanced, since the physiological transfer of electrons from one chain component to the other has been disturbed [25] (Figure 3B, WT+Anti.A). On the contrary, the uncoupler 2,4-dinitrophenol, which blocks respiration at the level of ATP synthesis, did not affect ROS production (Figure 3B, WT+2,4-DNP), as described elsewhere [25].

**Figure 4:**
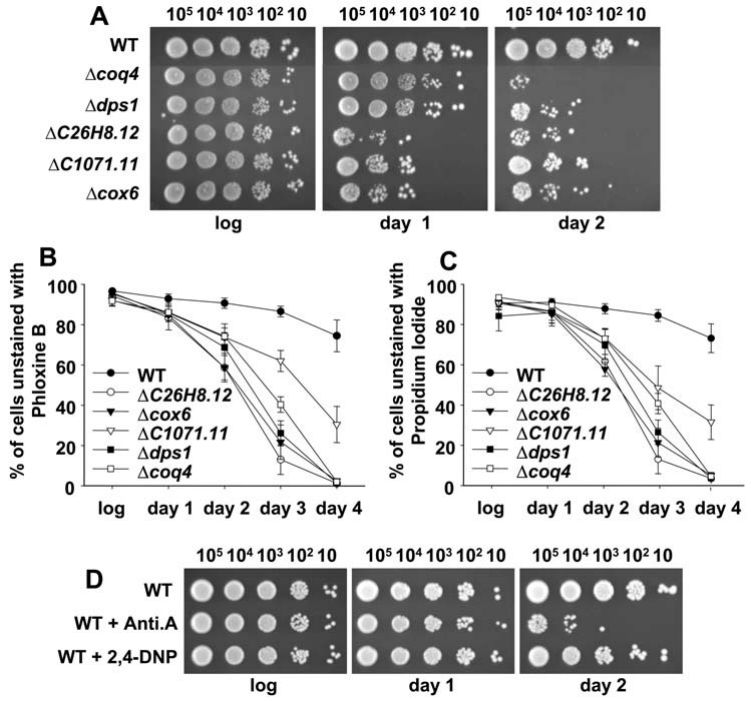
Inhibition of the electron transfer chain causes a reduction of the life span. (A) Respiratory mutants have reduced viability at stationary phase. Strains such as those in Figure 2A were grown aerobically in complex medium. The same number of cells (10–10^5^) were spotted onto complex media plates during the logarithmic phase (log), and 24 (day 1) and 48 hr (day 2) after reaching the stationary phase. (B, C) Mitochondrial mutants display lower percentages of metabolically active cells at stationary phase than wild-type cells. Samples as in A were incubated with phloxine B (B) or propidium iodide (C). The percentage of unstained cells was determined by flow cytometry. Data in both panels were obtained from 3–5 independent experiments and are expressed as mean±SEM. (D) Inhibition of respiration by antimycin A reduces viability during the stationary phase. Strain 972 (WT) was grown aerobically in defined medium in the presence or absence of antimycin A (WT+Anti.A) or 2,4-dinitrophenol (WT+2,4-DNP). The same number of cells (10–10^5^) were spotted onto defined media plates during the logarithmic phase (log), and at day 1 and day 2 after reaching the stationary phase.

We then tested the effect of intrinsic oxidative stress on the survival rates of chronologically aged cells. We compared the viability of wild-type and mitochondrial mutant cultures 24 and 48 hr after reaching stationary phase by spotting cells in complex media plates (Figure 4A), and the results indicate that the elevated levels of ROS in these mutants are clearly deleterious for cell survival. Measurement of the percentage of metabolically inactive cells, unable to efflux fluorescent dyes such as phloxine B (Figure 4B) or propidium iodide (Figure 4C), yielded similar results: short after entering stationary phase, the mitochondrial mutants display high percentages of metabolically inactive cells, when compared to wild-type cultures. The fitness of early stationary phase cultures of the mitochondrial mutants, prior to death, was also compromised, as determined by measuring protein carbonylation of logarithmic or stationary phase cultures (Figure S2). As indicated above, the use of electron transfer chain inhibitors such as antimycin A also decreases oxygen consumption and enhances electron leakage and ROS production (Figure 3B). Concomitantly, wild-type cells grown in the presence of antimycin A showed a decreased life span (Figure 4D; WT+Anti.A). The uncoupler 2,4-dinitrophenol, however, did not impair cell survival of wild-type cells (Figure 4D; WT+2,4-DNP), since it blocks ATP synthesis without an enhancement of ROS production (Figure 3B).

## Discussion

Exposure to ROS is an inevitable part of aerobic metabolism. Endogenous ROS is believed to be a source of chronic damage in aerobic organisms, since they can harm all biomolecules. Previous reports have shown that cells lacking sufficient levels of scavenging enzymes (eg, superoxide dismutases or peroxiredoxins) can display growth defects under aerobic conditions, since respiration of nonfermentable carbon sources would then raise ROS to toxic levels [26], [27]. We show here that increased production of ROS can also compromise growth in cells not devoid of antioxidant activities. We have isolated several strains lacking mitochondrial components that showed inhibited respiration and enhanced ROS production. Therefore, intrinsic oxidative stress can be achieved by genetic or drug-mediated disassembly of mitochondrial components and, specifically, by disruption of the mitochondrial electron transfer chain.

Double selection screening has proven useful when elucidating which of the strains with growth defects on defined medium suffer from oxidative stress and not from nutritional auxotrophies, since only the former group of strains would also display growth defects in complex medium in the presence of extracellular H_2_O_2_. Further proof that mitochondrial mutants suffer from oxidative stress is that they can grow in defined medium under anaerobic conditions, when respiration is minimal, or when cysteine or N-acetylcysteine are added. However, the growth defects of mitochondrial mutants are not fully reversed by adding exogenous cysteine to the cell cultures: lower cellular densities are reached during the stationary phase than with wild-type cells. This indicates that these mutants also suffer from inefficient utilization of glucose (only fermentation can take place). In fact, the deletion collection includes only a few other strains that are deficient in respiration and lack enzymes of the tricarboxylic acid pathway or mitochondrial ATPase. These strains do not show any deficiency during growth in defined media or complex media with extracellular H_2_O_2_, but they do reach lower cellular densities during the stationary phase and are unable to grow on nonfermentable carbon source glycerol (data not shown).

Electron leakage from the transport chain is probably enhanced, even though total oxygen consumption and ATP generation is severely inhibited in all the mitochondrial mutants. A similar effect has been described with electron transport chain inhibitors such as cyanide (inhibitor of complex IV) or antimycin A (blocks complex III) (for a review, see 28): by inhibiting the function of an electron carrier, these drugs cause the preceding carriers to accumulate in their reduced forms, which increases their rate of autoxidation and electron leakage [25]. However, we cannot rule out the possibility that mitochondrial dysfunction could trigger ROS formation at other cellular sites. Six of the 12 isolated strains lacking different components of the electron transfer chain are defective in ubiquinone synthesis (*dps1, coq2–5, coq10)*, which has been reported to have a role as a lipid-soluble antioxidant [29], [30]. In fact, these mutants that are defective in ubiquinone synthesis are more sensitive to intrinsic and extrinsic oxidative stress than other mitochondrial mutants (Figure 2A), which could be explained if ubiquinone deficiency both enhances ROS production and impairs cellular antioxidant capacity.

Our global screening, and the fact that the isolated mitochondrial mutants display increased ROS production, enhanced levels of oxidative stress, and reduced life span has important implications. Many studies suggest that the mitochondria has a basic role in aging and age-related neurodegenerative conditions such as Alzheimer's disease, Parkinson's disease, amyotrophic lateral sclerosis, and Huntington's disease [4], [31]. In many of these diseases, there is evidence that a decline in mitochondrial function occurs early in the course. Furthermore, studies using mutator mice, expressing proofreading-deficient versions of the mitochondrial DNA polymerase gamma, have shown that an increased rate of mitochondrial DNA mutations leads to decreased respiratory enzymatic activities and reduced ATP production, reduced lifespan, and premature onset of aging-related phenotypes, maybe due to enhanced ROS production (for a review, see 32). Furthermore, patients with nuclear-inherited isolated complex I deficiencies show a decrease in the amount of catalytically active complex I and increased rates of ROS production [33], [34], and a mutation in a component of complex II of *Caenorhabditis elegans* causes oxidative stress and aging [35]. However, whereas some of these reports emphasize the importance of reduced phosphorylation in the etiology of some of these diseases, others emphasize the primacy of oxidative stress (for a review, see 31). Our report provides a wider, more general view of the fact that a deficient electron transfer chain leads to basal oxidative stress and shorter life span in all aerobic cell types.

## Materials and Methods

### Yeast strains and growth conditions

We used the wild-type strains 972 (*h* ^−^), AV18 (*h* ^−^ *sty1*::*kanMX6*) [36], AV25 (*h* ^−^ *pap1*::*kanMX6*) [36] and EA38 (*h^−^ leu1 srx1::kanMX6*) [24]. Cells were grown in complex medium (also known as YE, with 3% glucose) or in defined medium (also known as synthetic minimal medium or MM; contains 2% glucose). When indicated, the 2% glucose of the defined medium was substituted by glycerol (in fact, 2% glycerol and 0.2% glucose). All these culture media were prepared as described elsewhere [22]. When indicated, 0.1 mg/ml cysteine or 1.6 mg/ml N-acetylcysteine was added as an antioxidant to liquid defined medium.

### Measurement of oxygen consumption

For oxygen consumption experiments, the cell cultures were first grown in their respective media to reach stationary phase, then diluted into fresh media at a concentration of 1.5×10^5^ cells/ml for cells grown in medium with glucose and of 7×10^5^ cells/ml for cells grown in medium with glycerol. Growth at 30°C continued for approximately 15–17 hr until cultures reached a final OD_600_ of 1. Two hours before harvesting the cells, the cultures were treated or not with 0.135 mg/l antimycin A (Sigma-Aldrich) or 25 mg/l 2,4-dinitrophenol (Sigma-Aldrich). Cells were washed once with the respective fresh media and, for the oxygen consumption assay, cells were diluted 1:10 in defined media (2% glucose) to a final OD_600_ of 1. The measurements were made using an HI9146 oximeter with an Hl7640714 probe (Hanna Instruments), and readings were recorded every minute for 15 min.

### H_2_O_2_ sensitivity assay and anaerobic growth conditions

It was performed as described elsewhere [27].

### Preparation of *S. pombe* extracts to measure protein carbonylation

It was performed as described elsewhere [27].

### High-throughput sensitivity screen

A haploid deletion collection of approximately 2700 non-essential *S. pombe* genes was obtained and will be described elsewhere. The collection was grown in liquid complex media (96 well plates) containing kanamycin (100 mg/ml) at 30°C for 2 days without shaking. Cultures were replicated with a 96-pin metal replicator (Sigma-Aldrich) on 4 types of solid plates: complex media with or without H_2_O_2_ (5 mM), defined media, or glycerol-containing media (defined media with 1% glycerol, 0.1% glucose). The plates were incubated at 30°C for 3–4 days.

### Measurement of intracellular H_2_O_2_ levels

Relative intracellular peroxide levels were analyzed using the redox sensitive fluorescent probe 2′,5′-dichlorofluorescein diacetate (DCFH-DA; Molecular Probes). This dye produces green fluorescence in the presence of H_2_O_2_ in both living and dead cells. To distinguish living cells from dead ones, we used a second indicator dye, propidium iodide (Sigma-Aldrich). Strains were grown in complex media to an OD_600_ of 0.3. When indicated, 0.135 mg/l antimycin A or 25 mg/l 2,4-dinitrophenol was added to the cell cultures two hours prior to harvesting. Cells were centrifuged, washed twice with phosphate buffered saline (PBS; 137 mM NaCl, 10 mM phosphate, 2.7 mM KCl, pH 7.4), and incubated with 50 µM DCFH-DA and 3 µg/ml propidium iodide for 40 min on ice in darkness. Peroxide steady-state levels and cell viability were simultaneously analyzed by flow cytometry. Propidium iodide was monitored in channel FL3 (red fluorescence-detecting), whereas DCFH-DA was monitored in channel FL1 (green fluorescence-detecting). Only cells negative for propidium iodide staining were analyzed for DCFH-DA-dependent green fluorescence. A total of 10,000 propidium iodide-negative cells were analyzed for each strain. For each cell culture, the absolute fluorescence numbers were normalized to cell size. Relative fluorescence values are indicated using the wild-type strain as a reference (with an assigned value of 1).

### Determination of survival rates of chronologically aged cultures

For the experiment with the mitochondrial mutants, strains were grown in complex media. For the experiment with mitochondrial inhibitors, wild-type cells were grown in defined medium, and the inhibitor was added or not to the liquid cultures at a final concentration of 0.135 mg/l antimycin A or 25 mg/l 2,4-dinitrophenol when cells were inoculated in fresh defined medium at an OD_600_ of ∼0.05. Cultures were incubated at 30°C until they reached stationary phase, at an approximate OD_600_ of 5–8, depending on the strains and growth conditions. The same number of cells in 5 µl were spotted on complex agar plates from cultures at the logarithmic phase (OD_600_ of ∼0.3) or 24 and 48 hr after reaching the stationary phase. The spots were allowed to dry and the plates were incubated at 30°C for 2–4 days. For viability tests using exclusion of fluorescent dyes as an indicator of metabolic activity, aliquots of the same cultures as above were stained with either propidium iodide or phloxine B. For propidium iodide staining, cells were centrifuged, washed twice with PBS, and incubated with 3 µg/ml of the dye for 40 min on ice in darkness. Regarding phloxine B, cells were incubated with 5 µg/ml of the dye for 2 h with shaking at 30°C in darkness, centrifuged and washed twice with PBS. For each sample, a total of 10,000 cells was analyzed by flow cytometry using channel FL3 for propidium iodide and channel FL2 for phloxine B.

## Supporting Information

Text S1 Supplementary Materials and Methods. (Zuin et al.)
(0.03 MB DOC)

Table S1 (Zuin et al.)
(0.07 MB DOC)

Figure S1 Activation of the Pap1 pathway requires lower H_2_O_2_ concentrations when the cells are grown in defined media. (A) Western blot analysis of the in vivo redox state of Pap1. Wild-type strain 972 was grown in complex or defined media and treated or not with H_2_O_2_ at the concentrations and times indicated. The redox state of Pap1 was analyzed by Western blot of TCA extracts (see Text S1). Reduced (inactive) and oxidized (active) Pap1 forms are indicated with arrows. (B) Northern blot analysis of the Pap1-dependent genes *tpx1* and *p25*. Total RNA from wild-type strain 972 was obtained from cultures of cells grown in complex and defined media and treated with H_2_O_2_ at the concentrations and times indicated (see Text S1), and probed against *tpx1*, *p25* and *cdc2* (loading control).
(3.49 MB TIF)

Figure S2 Figure S2. Protein carbonylation of logarithmic and early stationary phase cultures of wild-type cells and mitochondrial mutants. Wild-type strain 666 (WT-666), and its derivative deletion mutants *Δcoq4*, *Δdps1*, *ΔC26H8.12*, *ΔC1071.11* and *Δcox6* were grown aerobically in complex media. Cells were collected during the logarithmic phase (log) and 24 hr after reaching the stationary phase (day 1). Protein carbonylation was detected by reaction of carbonyl groups with DNPH, followed by SDS-PAGE and Western blot analysis by using anti-DNP antibody (α-DNP, top panels). As a control of strong protein carbonylation, wild type strain 972 (WT-972), grown logarithmically in complex media, was treated (1) or not (2) with 2 mM H_2_O_2_ for 30 min prior to protein extraction. Coomassie staining of the gels is presented as loading controls (Coomassie; bottom panels).
(4.78 MB TIF)
