# Supplementary material for: Mitochondrial Dysfunction Increases Oxidative Stress and Decreases Chronological Life Span in Fission Yeast
Source: PLoS One. 2008 Jul 30;3(7):e2842. doi: 10.1371/journal.pone.0002842 (PMC2475502; doi:10.1371/journal.pone.0002842)
Supplement: Text S1 — Supplementary Materials and Methods. (Zuin et al.) (0.03 MB DOC) [file pone.0002842.s001.doc]

**Text S1 (Zuin et al.)**

**Preparation of *S. pombe* TCA extracts and immunoblot analysis**. For *in vivo* redox state analysis of Pap1, TCA extracts were prepared as described elsewhere [1]. Immunoblotting was performed as described elsewhere [2]. Proteins were immunodetected using polyclonal anti-Pap1 antibody [3].

**RNA preparation for Northern blot analysis.** Cells grown in defined medium to a final OD_600_ of 0.5 were left either untreated or treated for 30 min with H_2_O_2_ at the indicated concentrations. RNA from each sample was then isolated and Northern blot performed as described elsewhere [3]. The blots were hybridized with [^32^P]dCTP-labelled *tpx1* and the Pap1-dependent *p25* probes. *cdc2* was used as a loading control.

1. Vivancos AP, Castillo EA, Biteau B, Nicot C, Ayte J, et al. (2005) A cysteine-sulfinic acid in peroxiredoxin regulates H2O2-sensing by the antioxidant Pap1 pathway. Proc Natl Acad Sci U S A 102: 8875-8880.

2. Zuin A, Vivancos AP, Sanso M, Takatsume Y, Ayte J, et al. (2005) The glycolytic metabolite methylglyoxal activates Pap1 and Sty1 stress responses in Schizosaccharomyces pombe. J Biol Chem 280: 36708-36713.

3. Vivancos AP, Castillo EA, Jones N, Ayte J, Hidalgo E (2004) Activation of the redox sensor Pap1 by hydrogen peroxide requires modulation of the intracellular oxidant concentration. Mol Microbiol 52: 1427-1435.
