# Supplementary material for: Mitochondrial Dysfunction Increases Oxidative Stress and Decreases Chronological Life Span in Fission Yeast
Source: PLoS One. 2008 Jul 30;3(7):e2842. doi: 10.1371/journal.pone.0002842 (PMC2475502; doi:10.1371/journal.pone.0002842)
Supplement: Table S1 — (Zuin et al.) (0.07 MB DOC) [file pone.0002842.s002.doc]

| **Table S1. 51 genes required for survival upon extrinsic (extracellular H**_2_**O_2_) and intrinsic (growth on defined medium) oxidative stress.** | |
| --- | --- |
| **NAME** | **FUNCTION** |
| **Mitochondrial function^a^** | |
| *C26H8.12* | Covalently the heme group to the apoprotein of cytochrome c |
| *C1071.11* | NADH dependent oxidoreductase; contains a flavin like domain |
| *rip1* | Subunit of cytochrome bc1, also known as respiratory complex III |
| *sco1* | Copper chaperone protein, essential for complex IV assembly |
| *C1672.04c* | High similarity to S.Cerevisiae cox19p; which is metal transport for complex IV assembly |
| *dps1* | Decaprenyl diphosphate synthase, required for ubiquinone biosynthesis |
| *cox6* | Heme A-containing chain of cytochrome c oxidase |
| *coq2* | Required for ubiquinone biosynthesis |
| *coq3* | Hexaprenyldihydroxybenzoate methyltransferase.Ubiquinone biosynthesis |
| *coq4* | Ubiquinone biosynthesis protein |
| *coq5* | C-methytransferase, ubiquinone biosynthetic process and aerobic respiration |
| *coq10* | Electron transport and cellular respiration.Ubiquinone biosynthesis |
| *C336.13c* | Removal of transit peptides required for targeting of proteins from mitochondrial matrix |
| *tom70* | Receptor that accelerates the import of all mitochondrial precursor proteins |
| *C8C9.06c* | Mitochondrial translation regulator , PPR domains |
| *mss1* | GTPase involved in 5-carboxymethylaminomethyl modification of mitochondrial tRNAs |
| *C2G2.07c* | Mitochondrial ribosomal protein (small subunit) |
| *C25B2.04c* | Mitochondrial ribosome assembly protein |
| *C1610.02c* | Mitochondrial ribosomal protein l1 |
| **Oxidative Stress^b^** | |
| *gcs1* | Glutamate-cysteine ligase, involved in synthesis of glutathione |
| *arg11* | N-acetyl-gamma-glutamyl-phosphate reductase/acetylglutamate kinase |
| *fep1* | Iron-sensing transcriptional regulator, involved in the regulation of iron transport |
| *cuf1* | Cu metalloregulatory transcription factor, involved in iron and copper homeostasis |
| *sua1* | Sulfate adenylyltransferase. Catalyzes the 1^st^ intracellular reaction of sulfate assimilation |
| *cys11a* | Cysteine synthase, involved in cysteine metabolism |
| *met11* | Methylenetetrahydrofolate reductase, involved in methionine metabolism |
| *met14* | Adenylylsulfate kinase, involved in sulfate assimilation |
| *met16* | Phosphoadenylyl-sulfate reductase activity, involved in methionine metabolism |
| *C4D7.06c* | Siroheme synthase, involved in methionine metabolism |
| **Transcription and chromatin regulation^c^** | |
| *C36.07* | Elongator subunit of RNA polymerase II |
| *C3H7.10* | Elongator complex, subunit Elp6 (RNA polymerase II transcription elongation factor) |
| *C10F6.08c* | Transcriptional regulator, HMG box |
| *dcr1* | Involved in G1 arrest and mating in response to nitrogen starvation |
| *arb2* | A member of argonaute siRNA chaperone. Required for histone H3 'Lys-10' methylation |
| *kap1* | Chromatin remodeling complex subunit Ngg1 (SAGA-like complex) |
| **RNA metabolism and translation^c^** | |
| *C1322.01* | 3'-5' exonuclease for RNA 3' ss-tail |
| *cpd1* | Subunit of DNA polymerase delta holoenzyme complex |
| *C3H8.09c* | RNA-binding protein, involved in packaging pre-mRNAs into ribonucleoprotein structures |
| *C25D12.06* | RNA helicase ATP-dependent |
| *C660.10* | Protein containing an elongation factor Tu GTP binding domain |
| **Others^c^** |  |
| *C18H10.11c* | Hypothetical protein, may be involved in RNA stabilisation |
| *C1635.01* | Voltage-dependent anion-selective channel |
| *mrs2* | Magnesium ion transporter |
| *C1071.02* | DNA repair protein, transcription from RNA polymerase II promoter |
| *mek1* | Cds1/Rad53/Chk2 family protein kinase, inhibits cdc25,required for meiotic recombination |
| *kin1* | Establishment and/or maintenance of actin cytoskeleton polarity |
| *asp1* | Acid phosphatase activity, important for the function of the cortical actin cytoskeleton |
| *sir1* | Sulfite reductase NADPH flavoprotein subunit |
| *ado1* | Adenosine kinase, involved in purine salvage |
| *C1539.03c* | Argininosuccinate lyase, involved in L-arginine biosynthesis |
| ^a^Genes related to the electron transport chain and other mitochondrial functions  ^b^Genes related to oxidative stress, such as metal homeostasis, glutathione, sulfur amino acid synthesis and cystein metabolism  ^c^Genes related to transcription, translation and other functions | |
