# Supplementary figures and images for: Mitochondrial Dysfunction Increases Oxidative Stress and Decreases Chronological Life Span in Fission Yeast
Source: PLoS One. 2008 Jul 30;3(7):e2842. doi: 10.1371/journal.pone.0002842 (PMC2475502; doi:10.1371/journal.pone.0002842)

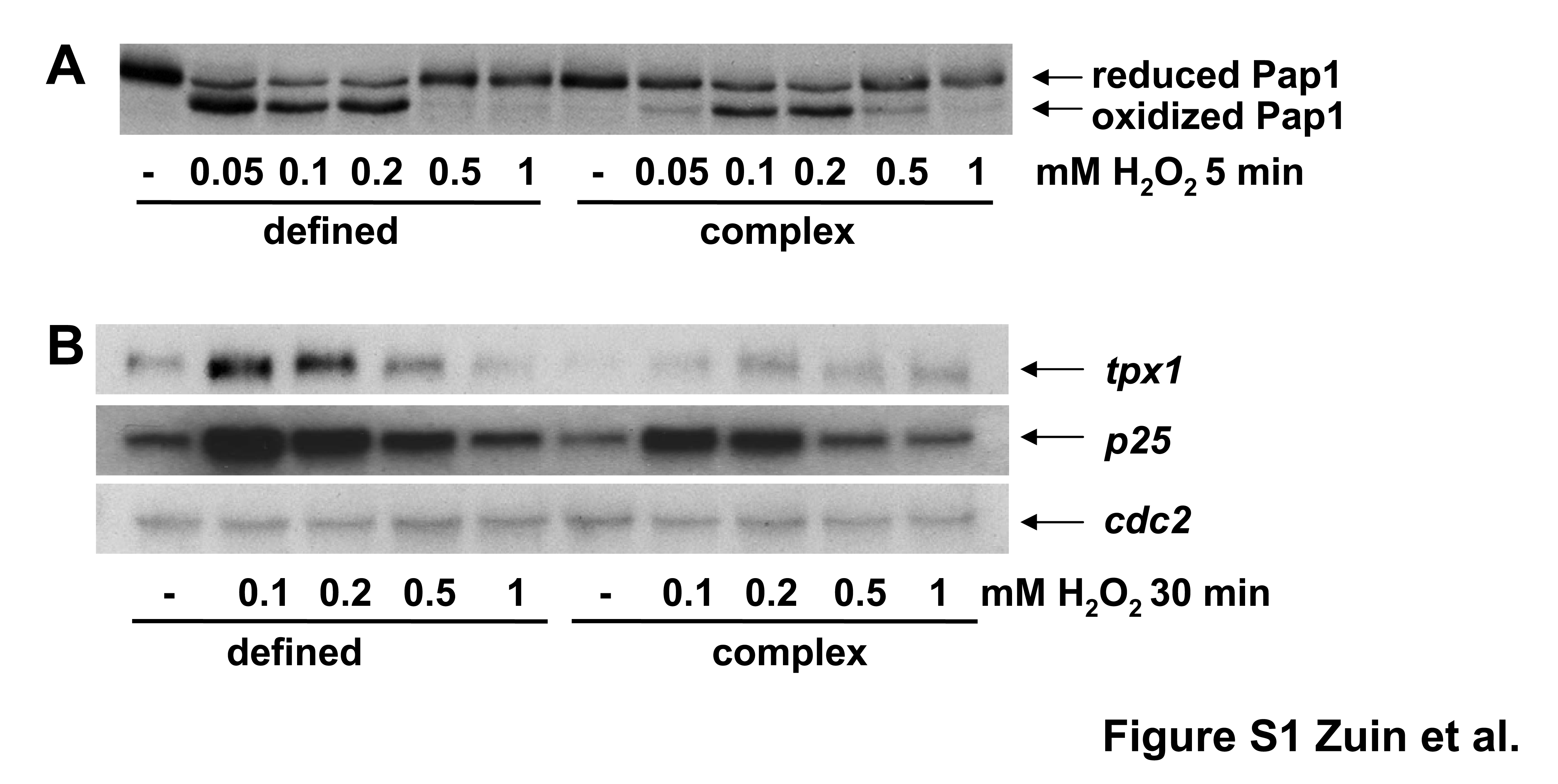

Supplement: Figure S1 — Activation of the Pap1 pathway requires lower H2O2 concentrations when the cells are grown in defined media. (A) Western blot analysis of the in vivo redox state of Pap1. Wild-type strain 972 was grown in complex or defined media and treated or not with H2O2 at the concentrations and times indicated. The redox state of Pap1 was analyzed by Western blot of TCA extracts (see Text S1). Reduced (inactive) and oxidized (active) Pap1 forms are indicated with arrows. (B) Northern blot analysis of the Pap1-dependent genes tpx1 and p25. Total RNA from wild-type strain 972 was obtained from cultures of cells grown in complex and defined media and treated with H2O2 at the concentrations and times indicated (see Text S1), and probed against tpx1, p25 and cdc2 (loading control). (3.49 MB TIF) [file pone.0002842.s003.tif]

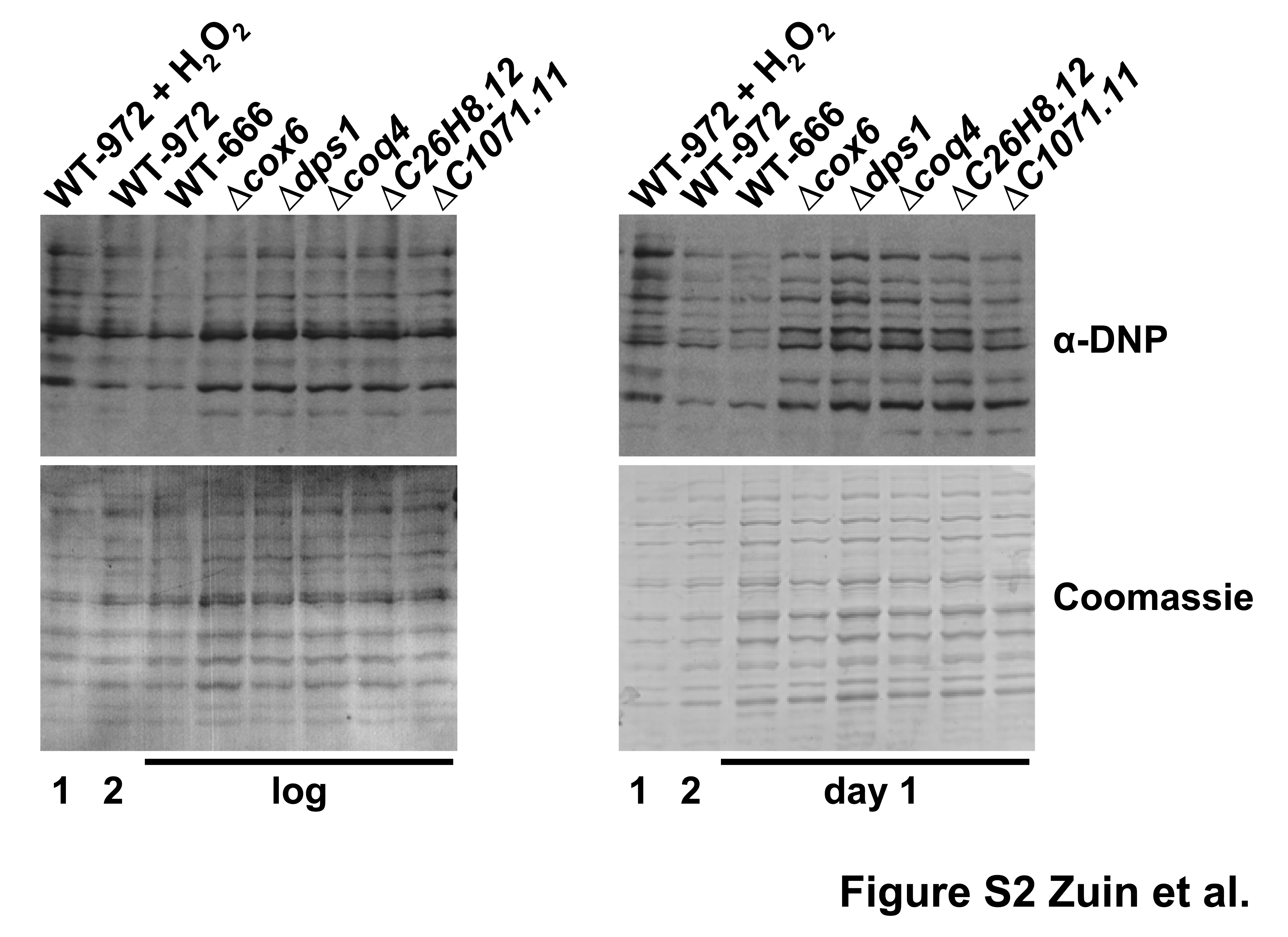

Supplement: Figure S2 — Protein carbonylation of logarithmic and early stationary phase cultures of wild-type cells and mitochondrial mutants. Wild-type strain 666 (WT-666), and its derivative deletion mutants Δcoq4, Δdps1, ΔC26H8.12, ΔC1071.11 and Δcox6 were grown aerobically in complex media. Cells were collected during the logarithmic phase (log) and 24 hr after reaching the stationary phase (day 1). Protein carbonylation was detected by reaction of carbonyl groups with DNPH, followed by SDS-PAGE and Western blot analysis by using anti-DNP antibody (α-DNP, top panels). As a control of strong protein carbonylation, wild type strain 972 (WT-972), grown logarithmically in complex media, was treated (1) or not (2) with 2 mM H2O2 for 30 min prior to protein extraction. Coomassie staining of the gels is presented as loading controls (Coomassie; bottom panels). (4.78 MB TIF) [file pone.0002842.s004.tif]
